# An effective model for the outpatient management of COVID-19

**DOI:** 10.1017/ice.2020.92

**Published:** 2020-03-26

**Authors:** Yuanyuan Xiao, Caixia Tan, Juping Duan, Anhua Wu, Chunhui Li

**Affiliations:** Xiangya Hospital of Central South University, Changsha, Hunan Province, China

*To the Editor*—A novel coronavirus, named SARS-CoV-2, has rapidly spread in China and multiple countries.^1,2^ Epidemiologically, SARS-CoV-2 is highly contagious.^3,4^ According to previous research, hospital-related transmission of SARS-CoV-2 was suspected in 41% of patients.^4^ In China, most people with a fever choose go to the hospital fever clinic as a top priority during this contagious period; therefore, the dense population of hospital outpatients is exposed to great underlying danger. In addition, these patients may have COVID-19 and/or other infectious diseases, such as seasonal influenza and including fever or respiratory symptoms of unknown origin. As a result, the risk of cross infection among these outpatients is extremely high. Thus, there is an urgent need for a reasonable patient screening and clearance process in the outpatient department.

The Xiangya Hospital of Central South University has much experience in screening and treating patients associated with COVID-19 and with reducing the risk of cross infection. First of all, we have strengthened the pre-examination and triage process. The attending physicians (or higher-status physicians) with rich experience in infectious diseases mainly take charge of screening patients, ensuring the accuracy of different triage procedures. Normally, however, the triage position is usually conducted by only 1 or 2 nurses. Second, after triage, patients are sent to the general clinic or the fever clinic according to their epidemiological history and clinical characteristics. The fever clinic is divided into 3 areas: (1) COVID-19 screening area for patients with suspected or identified COVID-19; (2) the common fever screening area, including patients with fever other than suspected COVID-19 or other respiratory diseases; and (3) the pediatric non–COVID-19 with fever screening area. These 3 areas are strictly separated, which can effectively prevent cross-infection among patients. Third, a consultation team consisting of doctors from the infectious disease, respiratory, and imaging departments has been implemented for timely consultation and screening of patients suspected to have COVID-19. Last but not least, healthcare workers are required to do a good job of self-protection, and we have optimized the distribution of personal protective equipment.

On January 30, 2020, the World Health Organization declared SARS-CoV-2 to be a Public Health Emergency of International Concern.^5^ On March 11, the WHO characterized COVID-19 as a pandemic.^6^ Other countries subsequently declared the COVID-19 outbreak an “emergency”—the highest warning tier, especially Italy, South Korea, and Iran, where a large-scale increase in the infection has occurred.^2^ To prevent the further spread of SARS-CoV-2 in these countries, emergency measures have been taken to manage outpatients. The practical diagnosis and treatment scheme for handling outpatients in Xiangya Hospital can provide guidance for other hospitals in China, as well as hospitals in other countries and regions around the world. More importantly, our strategy can provide an example for the prevention and control of infectious diseases in the future.
